# Effect of Roasting on the Chemical Composition and Oxidative Stability of Tomato (*Solanum lycopersicum* L.) Seed Oil

**DOI:** 10.3390/foods13111682

**Published:** 2024-05-27

**Authors:** Zhiya Niu, Zhongyan Zhu, Jing Zhou, Chengjian Xu, Changqing Wei, Wenyu Liu, Zhanxia Liu, Ting Wang, Hang Xiao

**Affiliations:** 1 Department of Food Science, College of Food Science and Technology, Sichuan Tourism University, Chengdu 610100, China; niuzhiya@stu.shzu.edu.cn (Z.N.); zhuzy@sctu.edu.cn (Z.Z.);; 2 School of Food Science and Technology, Shihezi University, Shihezi 832000, China; 3 Key Laboratory of Xinjiang Phytomedicine Resource and Utilization of Ministry of Education, Shihezi University, Shihezi 832000, China; 4 Institute of Agricultural Products Processing, Xinjiang Academy of Agricultural and Reclamation Science, Shihezi 832000, China; 5 Department of Food Science, University of Massachusetts, Amherst, MA 01003, USA

**Keywords:** tomato seed oil, roasting, chemical composition, oxidative stability index

## Abstract

In this study, tomato seed (TS) samples were subjected to different roasting conditions (90–170 °C and 10–30 min) to compare their effects on the chemical composition and oxidative stability of tomato seed oil (TSO). Unroasted TS was considered as a control sample. Our results revealed that moderate roasting (130 °C/20 min) can significantly increase the content of linoleic acid (54.01–54.89%), linolenic acid (2.17–2.41%), phytosterols (2789.56–3037.31 mg/kg), squalene (5.06–13.10 mg/kg), total phenols (22.37–22.67 mg GAE/100 g), and other functional components (*p* < 0.05) in TSO, while the antioxidant activity (via DPPH, ABTS, and FRAP assays) also increased. In addition, the tocopherol content decreased significantly (758.53–729.50 mg/kg). Accelerated oxidation experiments showed that roasting (170 °C/30 min) increased the oxidative stability index (OSI) of TSO from 5.35 to 7.07 h (*p* < 0.05). Furthermore, roasting gradually increased the content of 5-hydroxymethylfurfural (HMF) (0–1.74 mg/kg), which indicates that the oxidative stability and the degree of the Maillard reaction increased upon roasting. Principal component analysis (PCA) and hierarchical cluster analysis (HCA) showed that moderate roasting (130 °C/20 min) improved the chemical composition, antioxidant activity, and oxidative stability of TSO. Furthermore, this work provides a useful theoretical basis for the processing and wide application of TSO in the pharmaceutical and food industries.

## 1. Introduction

Tomatoes (*Solanum lycopersicum* L.) are one of the most important commercial and popular vegetables worldwide due to their desirable flavor and beneficial components [1,2]. In 2018, 1.82 × 10^8^ tons of tomatoes were produced globally, with a significant portion processed into juice, sauce, etc. During tomato processing, 3% of the total yield becomes a residue, resulting in an estimated 5.5 to 9.2 million tons of tomato pomace produced each year worldwide. Tomato seed (TS) is the main substance in tomato pomace. Currently, TS has been used in the formulation of dietary supplements. In addition, TS is utilized in functional cosmetics [3].

TS contains approximately 20% oil, composed of triacylglycerols (esters of fatty acids and glycerol), phenolic compounds, and other bioactive compounds such as tocopherols, sterols, and polar lipids, which are beneficial to human health [4]. Consequently, TS can play a positive role in health and nutrition [3,4,5]. Additionally, tomato seed oil has been developed and used as a substitute in bakery products and supplementary animal feeds due to its high protein content and other beneficial properties [6,7]. Moreover, its thermal stability and antioxidant potential make TS an effective food preservative agent [8]. In addition, extracting oil from TS helps reduce some of the environmental burden related to the tomato industry. Tomato seed oil (TSO) is of great interest due to its healthful and nutritive effects, including antioxidant, antitumor, and antibacterial activities, as well as its provision of essential fatty acids, such as linoleic acid [5,9]. Nowadays, there has been growing interest in special vegetable oils like TSO because of their phytochemicals and potential health benefits.

Cold pressing is a widely used extraction method for conventional and secondary edible oils. In general, cold-pressed oils have better organic qualities [10] because they are obtained via mechanical pressing, making them free of chemicals and safer than the traditional solvent-extraction methods. Compared to oil recovery using solvents, cold pressing is also simpler as it involves fewer steps [10,11]. In recent years, there has been an increasing trend for commercially available cold-pressed oil. Despite its popularity, cold pressing has a low oil extraction efficiency. To overcome this issue, a novel thermal pretreatment step is commonly applied to oil seeds prior to pressing. Heat treatment shows a significant effect on enzymes. For instance, undesirable enzymes (predominantly lipases) can be deactivated during heating. Moreover, heat treatment can also increase the oil yield [11]. Moderate roasting before cold pressing produces high antioxidant activity and tocopherol levels in TSO. This pre-treatment does not impact TSO’s fatty acid and mineral content but maximizes oil yield [12].

Roasting is a traditional processing method for generating aroma-enriched flavored oils, which increases the availability of nutrients and deactivates undesirable enzymes in the final oil product. Several thermal and chemical reactions confer a desired flavor and color to roasted seeds, which enhances the palatability of the oil [13]. Roasting affects the phenolic content and antioxidant activity of the oil, and the oxidative stability of various oils, including safflower, pine nut, tart cherry kernel, and pumpkin seed oils, has been reported to be enhanced upon roasting and is attributed to the formation of Maillard reaction products (MRPs) [14,15,16]. However, the roasting process also has negative effects. If it is not properly controlled, roasting can lead to adverse outcomes, such as the production of off-flavors and toxic compounds (acrylamide) [17,18].

These findings indicate that the overall quality of TSO can be enhanced under appropriate roasting conditions. To date, there have been only a few studies on TSO with some research discussing the influence of different extraction techniques on its physicochemical properties [19]. In addition, there are no research studies reported on TSO related to the roasting-screw pressing method. Consequently, our study aimed to assess the effect of roasting TS at different temperatures (90, 130, and 170 °C) for various durations (10, 20, and 30 min) on the physicochemical properties, phenolic compounds, fatty acids, and tocopherol contents, oxidative stability index, and antioxidant capacity of TSO. These specific temperatures and treatment durations were chosen with the aim of improving the quality of TSO through moderate heating, as combining the advantages of cold pressing and hot pressing was not feasible. Furthermore, this approach seeks to reduce the energy consumption in the industrial production of TSO by optimizing the heating time and method. Our objective was also to obtain the correlations between TS roasting methods and the quality of TSO and to discover the optimal roasting conditions using statistical techniques.

## 2. Materials and Methods

### 2.1. Materials

#### 2.1.1. Raw Materials

TS isolated from Xinfan 85 tomato was kindly supplied by Xinjiang Baihejing Biotechnology Co., Ltd. (Xinjiang, China); it was collected from Xinjiang Province and harvested during the 2020–2021 season. TS was handpicked, and seeds with blemishes, defects, and insect damage were removed; the remaining seeds were washed with tap water to remove impurities. A total of 9 kg of TS was divided randomly into 30 groups of 300 g each. Of these, 27 portions were subjected to roasting, while the remaining 3 portions were used as blank controls and stored at 25 °C.

#### 2.1.2. Chemicals

Palmitic, stearic, oleic, linoleic, and linolenic acid standards, 2,2-diphenyl-1-picrylhydrazyl (DPPH), 2,2′-azino-bis (3-ethylbenzothiazoline-6-sulfonic acid) diammonium salt (ABTS), and 2,4,6-tris (2-pyridyl)-1,3,5-triazine (TPTZ) were purchased from Sigma-Aldrich Inc. (St. Louis, MO, USA) and Sigma Aldrich Chemical Co., Ltd. (Shanghai, China). Other solvents and reagents were purchased from Tianjin Fuyu Fine Chemical Co. (Tianjin, China).

### 2.2. Roasting and Oil Extraction

TS was roasted using an automatic electric roaster at different temperatures (90, 130, and 170 °C) under continuous agitation for 10, 20, and 30 min per temperature. After roasting, the roasted TS was fed into a screw press (ZY22A, Wenzhou Hongkuo Technology Co., Ltd., Wenzhou, China) to extract the TSO. The relevant parameters of the screw press were capacity 3.5–4 (kg/h), motor power 500–750 (kw), screw speed (frequency) number 300 (r/min), opening size (nozzle) 50 (mm), and outlet oil temperature 30 °C. During the screw pressing step, the cloudy oil was subjected to centrifugation at 8000 rpm for 15 min at 4 °C. The TSO was then transferred directly into 100-mL dark colored bottles and stored at 4 °C in the dark. All samples were produced in triplicate.

### 2.3. Determination of the Physicochemical Properties of TSO

The physicochemical properties of TSO, including the acid value (AV) and peroxide value (POV), were characterized using the official AOCS methods Cd 5a-40 and Cd 8-53, respectively [20].

### 2.4. Determination of the Fatty Acid Composition (FAC) of TSO

The FAC of TSO was determined using gas chromatography (GC; Agilent 7890B, Santa Clara, CA, USA). The gas chromatograph was equipped with a flame ionization detector (FID) (Shimadzu, Kyoto, Japan), and a capillary HP-88 column (100 m × 0.25 mm × 0.20 μm) was used to separate the fatty acid methyl esters. The instrumental conditions were carried out in accordance with a published method with minor modifications [21]. The oven temperature program was 120 °C (1 min), followed by ramping at 2 °C/min up to 140 °C, (5 min) and 4 °C/min up to 230 °C (10 min). The temperatures of the injector and detector were 230 and 280 °C, respectively. The injection volume and separation ratio were 1 μL and 50:1, respectively. High-purity nitrogen (N_2_) was utilized as the carrier gas at a flow rate of 1 mL/min. FID was performed with hydrogen (H_2_) and air at 30 mL/min and 400 mL/min, respectively. The retention times were determined using fatty acid methyl ester standards. Standard solutions including stearic acid, palmitic acid, oleic acid, linoleic acid, and linolenic acid standard master batch (1.0 mg/mL) were prepared at different concentrations (0.2, 2, 6, 10, and 25 µg/mL). Standard curves were obtained by regression analysis, and the regression equations for each fatty acid are shown below:

Stearic acid: Y = 21222X − 4357.8 (R^2^ = 0.9900), (1)

Palmitic acid: Y = 19258X − 3057 (R^2^ = 0.9909), (2)

Oleic acid: Y = 19913X + 5668.5 (R^2^ = 0.9901), (3)

Linoleic acid: Y = 16056.6X + 45264 (R^2^= 0.9908), (4)

Linolenic acid: Y = 22015X − 5468 (R^2^= 0.9919), (5)

where X is the concentration of the standard solution and Y is the peak area.

In order to effectively compare the fatty acid distribution of each treatment, the results are presented as the total fatty acid percentage.

### 2.5. Determination of the Tocopherol Content of TSO

The tocopherol content was determined using HPLC, as described in a previous study with slight modifications [22,23]. TSO (1 mL) was extracted with 5 mL of methanol three times. The qualitative and quantitative identification of tocopherols was performed using high-performance liquid chromatography (HPLC; LC-20AT, Tokyo, Japan) equipped with a UV detector (SPD-20A, Shimadzu, Tokyo, Japan) and Eclipse Plus C_18_ column (250 × 4.6 mm, 5 μm, Agilent Technologies, Santa Clara, CA, USA). The mobile phase was n-heptane/tetrahydrofuran (98/2, *v*/*v*) with a column temperature of 35 °C, flow rate of 1.0 mL/min, sample volume of 10 μL, and elution wavelength set at 295 nm. Standard solutions of α, γ, and δ tocopherol standard master batch (1.0 mg/mL) were prepared in methanol (chromatographic grade) as mixed standard solutions at different concentrations (0.2, 0.5, 1, 2, 4, 6, 8, and 10 µg/mL). Standard curves obtained using regression analysis and the regression equations for each tocopherol are shown below:

α-tocopherol: Y = 10228X + 1234.1 (R^2^ = 0.9994), (6)

γ-tocopherol: Y = 18757X + 9475.7 (R^2^ = 0.9992), (7)

δ-tocopherol: Y = 11583X − 7180.8 (R^2^ = 0.9994), (8)

where X is the concentration of the standard solution and Y is the peak area.

Each tocopherol was identified by comparing its peak retention time with the reference standard, and the content is reported in mg/kg.

### 2.6. Determination of the Phytosterols and Squalene Contents of TSO

The squalene content was determined using gas chromatography–mass spectrometry (GC-MS), as described in a previous study with slight modifications [24]. Phytosterols and squalene analyses were performed using a GC-MS system coupled to an FID (Thermo Fisher, Waltham, MA, USA) equipped with a DB-5 capillary column (0.25 μm, 30 m × 0.25 mm, Agilent, Santa Clara, CA, USA). The operating temperature conditions were as follows: 150 °C for 4 min, increase to 270 °C at 10 °C/min, and maintain at 270 °C for 18 min. Helium was used as the carrier gas at a flow rate of 1 mL/min; the split ratio was 1:10, and the mass range was 50–550 m/z. The master standard solution of 5α-cholestane (1.0 mg/mL) was prepared with methanol (chromatographic grade) as mixed standard solutions of different concentrations (2, 5, 10, 15, and 20 µg/mL). The standard curves were generated by regression analysis, and the regression equation of 5α-cholestane is shown below:

Y = 8075.2X + 428.99 (R^2^ = 0.9989), (9)

where X is the concentration of the standard solution and Y is the peak area.

5α-Cholestane was used as a reference standard for the determination of the phytosterols and squalene in TSO; the results are presented in mg/kg.

### 2.7. Determination of the Hydroxy Methyl Furfural (HMF) Content of TSO

The determination of HMF was performed according to a published method [24] using an HPLC (LC-20AT, Shimadzu, Tokyo, Japan) system equipped with a UV detector (SPD-20A, Shimadzu), binary pump, and EclipsePlus C18 column (250 × 4.6 mm, 5 μm, Agilent Technologies).

### 2.8. Determination of the Non-Enzymatic Browning Index (BI) of TSO

The non-enzymatic browning index (BI) of TSO was measured according to the method of Cai et al. [17]. Solutions were acquired at an oil to chloroform ratio of 1:20 (*w*/*v*), and the BI (absorbance at 420 nm) was measured using a spectrophotometer.

### 2.9. Determination of the Total Polyphenol Content of TSO

The total polyphenol content (TPC) was estimated spectrophotometrically using Folin–Ciocalteu reagent, as described in a previous study [25,26]. In this procedure, the sample (250 mg) was hydrolyzed at room temperature for 4 h using 5 mL of 4 mol/L NaOH solution. Following the hydrolysis step, the pH of the mixture was then adjusted to 2.0 with 6 mol/L hydrochloric acid, and the hydrolysate was extracted three times with n-hexane. The combined extracts were ethanolized under a stream of nitrogen, and the residue was reconstituted in 1.5 mL of methanol–water (50:50, *v*/*v*). In the same way, the TSO was extracted three times with methanol–water (70:30, *v*/*v*) to obtain the TPC of the TSO samples. The extracts were blended with the Folin–Ciocalteu reagent, which was diluted in aqueous distilled water (1:10, *v*/*v*) and 20% Na_2_CO_3_. The samples were left in the dark for 2 h, concentrated by centrifugation at 7155× *g* for 2 min, and the absorption of the supernatant was measured at 765 nm using a UV–Vis spectrophotometer (Shimadzu, Kyoto, Japan). The standard calibration curves were prepared using various concentrations of gallic acid (0–100 μg/mL), and the TPC was measured as gallic acid equivalents (GAE) for each liter of oil and 100 g of seeds.

### 2.10. Determination of the Antioxidant Capacity of TSO

The methanolic extract of TSO was prepared following the analytical method used to determine the HMF content. The scavenging activities of the methanolic extract towards DPPH, ABTS, and FRAP were based on the standard profile of Trolox (linear range 5–50 μmol/L) and represented as micro-moles of Trolox equivalent (TE) for each 100 g of oil (μmol TE/100 g). The procedure was based on a previous study reported by Ballus et al. [27].

### 2.11. Determination of the Oxidative Stability of TSO

The oxidative stability index (OSI) was measured using an OSI-24 Rancimat apparatus (Omnion, FL, USA). Three grams of TSO was treated in a reaction tube containing 50 mL of distilled water and heated to 110 °C with an in-flow rate of 10 L/h. The results were estimated and recorded in hours (h) manually by the instrument software.

### 2.12. Statistical Analysis

All the data are presented as the average ± standard deviation of the analysis conducted in triplicate. Analysis of variance (ANOVA) and Duncan’s test were performed using Statistical Products and Services Solution (SPSS version 25.0) software to assess the differences, and *p* < 0.05 was considered statistically significant.

## 3. Results and Discussion

### 3.1. Acid Value (AV)

**Figure 1:**
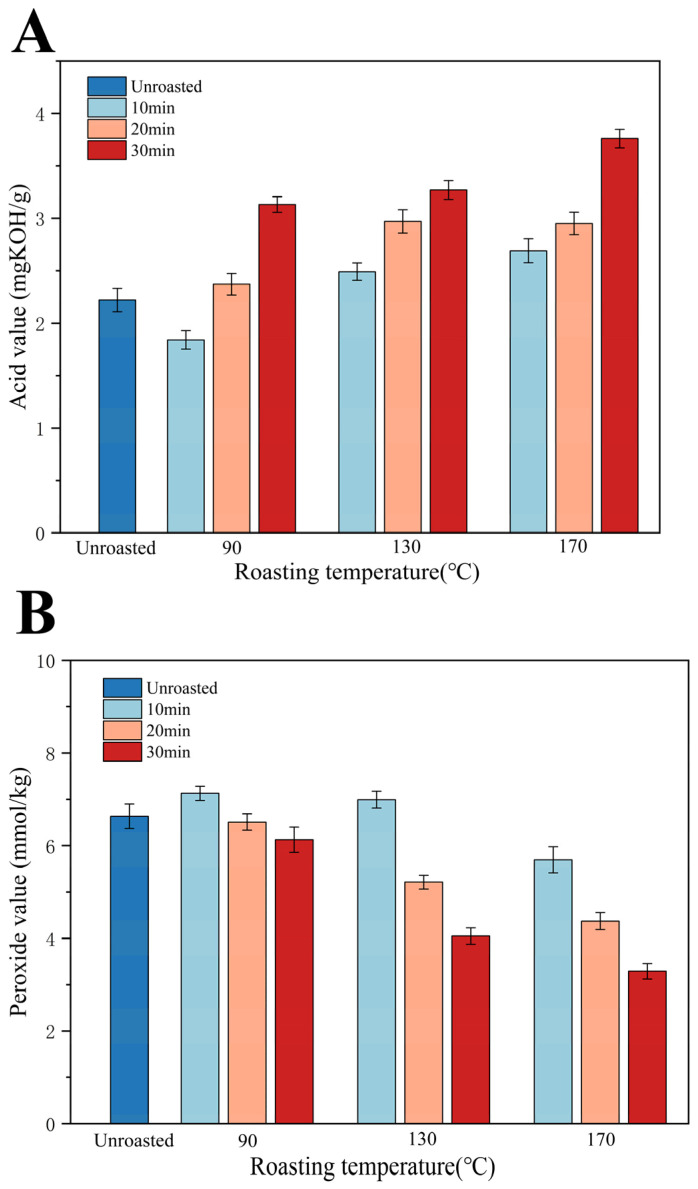
Effect of roasting on the AV (**A**) and POV (**B**) of TSO at 90, 130, and 170 °C for 10, 20, and 30 min.

The influence of roasting time and temperature on the AV of TSO is presented in Figure 1A. Oil oxidation, which occurs through complex processes, can be classified into enzymatic-oxidation, photo-oxidation, and auto-oxidation, which are the primary pathways of oil oxidation. During this process, the oxidation and degradation of fatty acids occur. Consequently, the free FA content can be used as an important indicator to evaluate the degree of oxidation in TSO.

The AV mainly characterizes the concentration of FA produced via oxidation and the rancidity of oil. Our results revealed that the AV of TSO increased during the roasting process and ranged from 1.84 to 3.76 mg KOH/g (Figure 1A). According to the Codex Alimentarius Commission (CAC), the AV of virgin oil should not be more than 4 mg KOH/g, and all of the obtained data were below this specified range. The increase in AV may be associated with the increase in the roasting temperature; thermal treatment accelerates the hydrolysis reaction of triglycerides in TSO, leading to an increase in the free FA content. Similar results have been reported by Wroniak, in which roasting at 150 °C resulted in an elevated degree of hydrolysis of rapeseed oil [28]. In contrast, Zou et al. have reported that the AV of wheat germ oil decreases rapidly due to the inactivation of oxidase at higher temperatures [29].

### 3.2. Peroxide Value (POV)

The effects of the roasting time and temperature on the POV of TSO are presented in Figure 1B. The POV reflects the content of primary oxidation products in oil, which are formed from the unsaturated esters present in oil during the oxidation process.

Figure 1B shows that the POV of TSO initially increased and then decreased upon roasting. According to the CAC, the POV of virgin oil should not be more than 7.5 mmol/kg, and all of the data in the current study were below the specified range. The POV of TSO obtained from the unroasted TS was 6.63 mmol/kg, and the POVs of all the TSO samples were below the limit (POV < 7.5 mmol/kg). The POV of the TSO extracted from the roasted TS gradually increased until the temperature reached 130 °C for 10 min (7.13 mmol/kg) and then decreased to 3.29 mmol/kg (170 °C/30 min). These results may be attributed to the decomposition of hydroperoxide, which was accelerated at high roasting temperatures to form aldehydes, ketones, and acids. Similar variations in the POV of oil obtained from sesame seeds after roasting have been reported in a previous study [30].

### 3.3. Oil Yield

**Figure 2:**
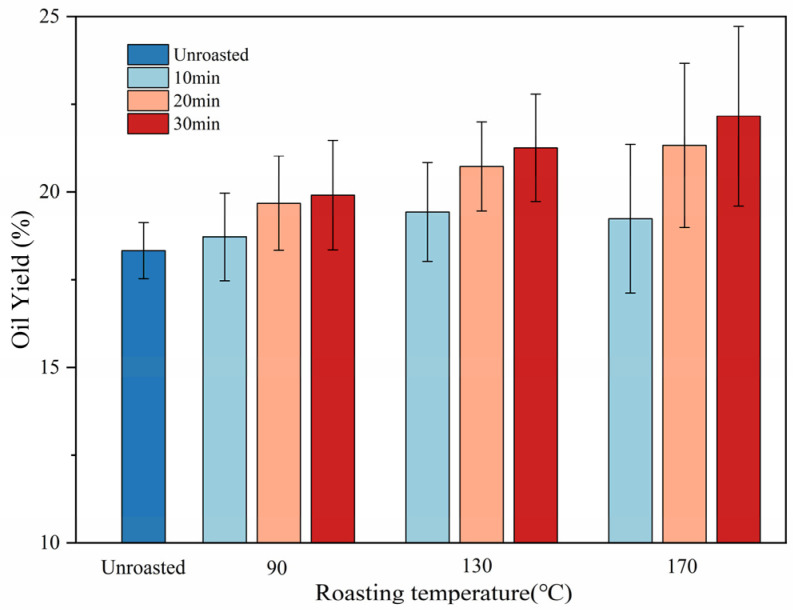
Effect of roasting on the oil yield of TSO roasted at 90, 130, and 170 °C for 10, 20, and 30 min.

The roasting process resulted in changes in the TSO oil yield, as shown in Figure 2. The oil yield of TSO showed an overall upward trend with increasing roasting temperature and time. The original oil content of the TSO obtained from raw unroasted TS was 18.72%. The percentage of TSO extracted from seeds markedly increased (*p* < 0.05) as the roasting temperature (up to 170 °C) and time (up to 30 min) were increased, rising from 18.72 to 22.16%. This phenomenon could be attributed to the thermal treatment process, which promoted oil release by decreasing enzyme activity, coagulating proteins, and inducing structural changes [11,16]. In this study, moderate roasting was employed, using different temperature and time parameters from those applied in hot pressing, to investigate how the overall quality of TSO can be improved by moderate roasting.

### 3.4. Maillard Reaction Products (MRPs)

**Figure 3:**
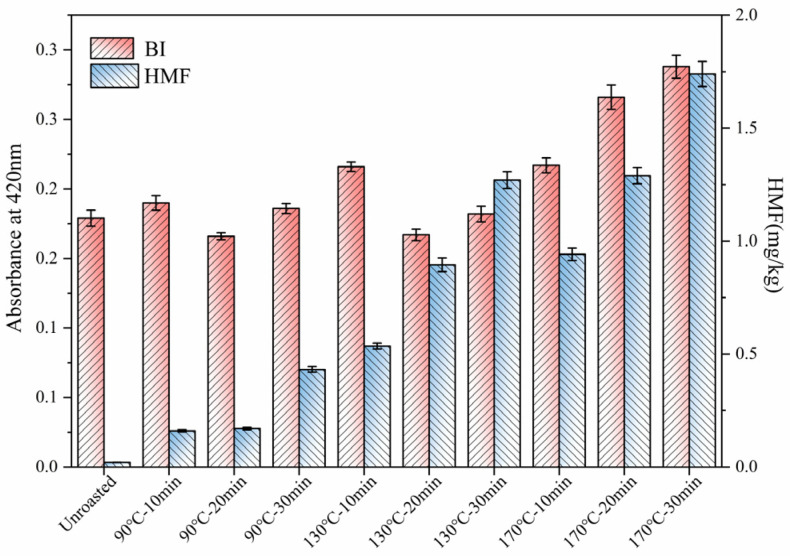
Effect of roasting on the MRPs of TSO.

To elucidate the passage of MRPs to the oil phase, the changes in the non-enzyme browning index and HMF, an intermediate formed during thermal processing, were determined [31]. The effects of roasting on the BI and HMF are given in Figure 3. The BI of TSO was gradually increased with an increase in the roasting temperature and time. The highest absorbance at 420 nm was observed in TSO obtained from TS roasted at 170 °C for 30 min. A similar finding has also been observed in wheat germ roasting [29]. Our analysis showed that no HMF was detected in TSO obtained from unroasted TS, and the highest content of HMF (1.74 mg/kg) was observed in TS roasted at 170 °C for 30 min, which indicates that roasting can promote the production of MRPs of TSO. The BI exhibited a positive correlation with HMF and can be regarded as an indicator of the severity of a heat treatment process for various foods.

### 3.5. Fatty Acid Composition (FAC)

The effects of roasting on the FAC of TSO observed using chromatography are shown in Table 1. The most predominant fatty acid was linoleic acid (C18:2) (53.80–55.59%), followed by oleic acid (C18:1) (22.58–23.07%), and palmitic acid (C16:0) (13.26–13.97%). These fatty acids are known for their broad range of biological activities and health benefits [32]. Our results are similar to those for previously reported fatty acids in TSO [8]. The fatty acids in TSO vary slightly with the roasting temperature and time. Statistical analysis indicated an increase in the SFA content and a slight decrease in the PUFA content in TSO obtained from roasted TS. This slight reduction may be attributed to the degradation of unsaturated fatty acids in the oil at elevated roasting temperatures. Similar results have been reported in previous studies on black cumin and sesame seeds [33,34]. Interestingly, Chirinos et al. compared cold-pressed *plukenetiahuayllabambana* seed oil extracted at different roasting temperatures and found insignificant variations in the fatty acid content across different treatments [35].

### 3.6. Tocopherol Contents

TSO contains high levels of tocopherols, which play a vital role as antioxidants in disease prevention. The variation in the individual tocopherol isomers and total tocopherols in various TSOs is listed in Table 2. TSO contains three types of tocopherols: α-tocopherol (7.76–31.82 mg/kg), γ-tocopherol (446.89–535.32 mg/kg), and δ-tocopherol (163.71–213.52 mg/kg). More than 85% γ-tocopherol and δ-tocopherol was found in TSO when compared to other vegetable oils [36]. Schmidt reported that the antioxidant activity of tocopherols in the lipid system was in the following order: γ > δ > α > β [37]. Therefore, the high ratio of γ-tocopherol and δ-tocopherol contributes to TSO’s protective antioxidant properties. The content of tocopherols in TSO extracted from roasted TS was slightly lower, but it is worth noting that α-tocopherol was positively affected by the roasting process. δ-Tocopherol changed in a similar manner to γ-tocopherol, both of which showed a noticeable decrease at 170 °C or after roasting for 30 min. The total tocopherol content was reduced by 15.1% after roasting compared to raw TSO, which might be attributed to the thermal degradation at an elevated temperature. Similar results have been reported for wheat germ, which was heat treated at 180 °C for 0–20 min [32]. In contrast, heat treatment has been previously reported to enhance the extraction of tocopherol from several oil seeds and nuts by disrupting the cell structure and increasing the extractability of tocopherol [38,39]. Our results showed that while roasting led to a slight decrease in the total tocopherol content, α-tocopherol was significantly increased compared to the control group.

### 3.7. Phytosterol Content

Phytosterols, primarily derived from vegetable oils, play a significant role in reducing blood cholesterol and lowering the risk of cardiovascular diseases. The roasting conditions can significantly affect the phytosterol content in TSO (Table 2). β-Sitosterol is the major component (1584.94–1788.64 mg/kg), accounting for more than 55% of the total phytosterols. Compared with the control group, the β-sitosterol content was significantly increased at 90 °C for 10–20 min and 130 °C for 10–30 min but decreased slightly with more intense heat treatment. The content of Δ5-avenosterol increased under all of the roasting conditions studied when compared to the control with the conditions of 10–30 min at 130 and 170 °C being the most favorable. In contrast, the contents of stigmasterol, cholesterol, and campesterol decreased significantly at 170 °C. Overall, the sum of the six phytosterols showed significant changes as the roasting temperature increased, with higher contents observed upon heating at 90 °C and 130 °C for 20 min. The results obtained for the phytosterol content were similar to those reported for cold-pressed rapeseed oil from roasted seeds [40] but contrasted with the results observed for the lipid fraction of cocoa beans [41]. This difference may be attributed to the change in the humidity during seed roasting, which can facilitate the extraction of phytosterols.

### 3.8. Squalene Content

Squalene is a natural antioxidant with strong biological activity, commonly found in marine life and vegetable oils. The squalene content in TSO obtained from roasted seeds is presented in Table 2. During the roasting process, the squalene content in TSO first increased and then decreased with the highest content (13.10 mg/kg) obtained after roasting at 130 °C for 20 min. Tikekar found that 12% of squalene in the oil-rich part of amaranth was reduced during the roasting process (150 °C, 20 min) [42]. Our results indicate that heat treatment increased the squalene content in TSO, which can be attributed to the release of fatty and fat-soluble compounds as a result of the disruption of the cell membrane of the oil body [43].

### 3.9. Total Polyphenol Content (TPC)

The variation in the TPC of TSO obtained from TS prepared under different roasting conditions is shown in Table 2. The TPC of the control was 22.95 mg GAE/100 g, which was substantially enhanced as the roasting temperature increased, peaking at 26.67 mg GAE/100 g obtained at 130 °C for 20 min. However, TPC then decreased progressively to 22.37 mg GAE/100 g as the temperature rose to 170 °C. Thus, TSO obtained from roasted TS has a higher content of phenolic compounds, which may be attributed to the denaturation of the proteins associated with these phenolic compounds, making them easier to extract [25]. Moreover, roasting allows the release of polyphenols bound to various cellular components, leading to an increase in the polyphenolic content. The subsequent reduction in the polyphenol content may be due to their thermal degradation at high temperatures.

### 3.10. Oxidative Stability Index (OSI)

The OSI is an important parameter used to evaluate the resistance ability of oils toward oxidative degradation. Table 3 shows that the OSI of TSO obtained from unroasted TS is 5.35 h and that as the roasting process progressed, the OSI of TSO decreases slightly during the initial stage (0–10 min) and then increases significantly (10–30 min). The significant overall increase in the OSI value of TSO with temperature and time is probably due to the compositional changes in fatty acids. Oxidation and degradation of PUFAs occurred during roasting, resulting in decreased PUFAs and increased SFAs, which led to an improved OSI. The OSI of TSO obtained from TS roasted at 170 °C for 20–30 min decreased slightly with prolonged roasting time. Our results confirmed that TSO has high thermal stability (5.35–7.36 h) and that roasting considerably enhanced the OSI value, which may be due to the generation of MRPs during the roasting process and the synergistic antioxidant effect of lipid nutrients (tocopherols, squalene, and sterols) [41,42].

### 3.11. Antioxidant Capacity

The results of our TSO antioxidant capacity measurements (DPPH, FRAP, and ABTS) are listed in Table 3. The DPPH values ranged from 43.17 to 59.78 μmol TE/100 g. In the FRAP assay, the results ranged from 36.39 to 44.78 μmol TE/100 g. For ABTS, the data were in the range of 48.23–59.95 μmol TE/100 g. Our statistical analysis disclosed that roasting results in statistically significant differences in the levels of antioxidant activity of TSO. In particular, the DPPH of the unroasted TSO was 43.17 μmol TE/100 g, which was comparable to the results reported by Özbek (the DPPH-radical scavenging activity of the unroasted tomato seeds, 1300 mmol TE/kg) [43]. The control sample presented the highest value in the FRAP test but had lower values in the DPPH and ABTS assays. The DPPH, ABTS, and FRAP assays displayed marked increases upon roasting at 90–170 °C for 20–30 min when compared to the control with a slight change after 10 min of roasting. Roasting leads to an increase in the antioxidant capacity, which is consistent with our oxidative stability results. This is probably due to the fact that roasting at high temperatures breaks down the chemical bonds in the cell matrix, making the molecules more soluble and enhancing the liberation of bioactive substances that contribute to antioxidant activity [44].

### 3.12. Principal Component Analysis (PCA)

**Figure 4:**
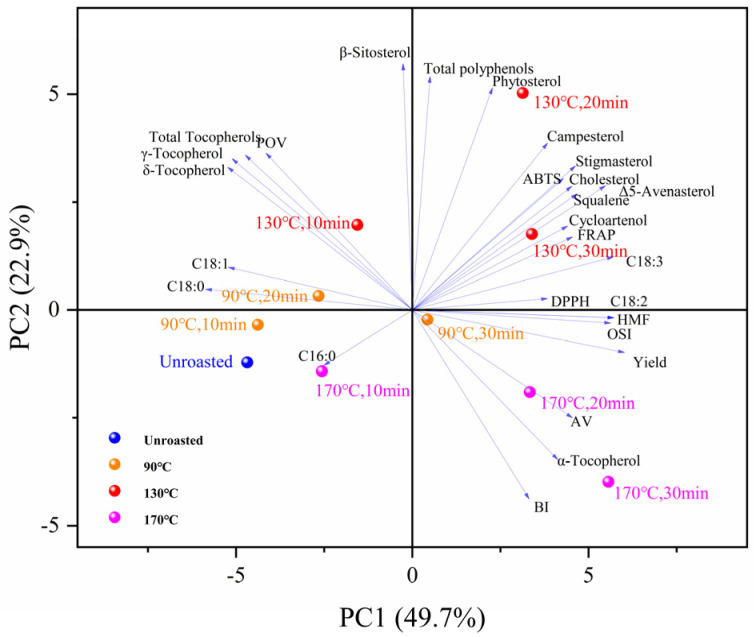
PCA. Note: C16:0—palmitic acid; C18:0—stearic acid; C18:1—oleic acid; C18:2—linoleic acid; and C18:3—linolenic acid.

Multivariate analysis was conducted using IBM SPSS Statistics 25 software to identify the correlations among the various physicochemical properties, tocopherol, phytosterols, oxidative stability, antioxidant activity, FAC, and MRPs of TSO obtained from unroasted and roasted TS. A total of twenty-seven variables, with three replicates (*n* = 3) were included in our statistical evaluation. The loading plot of the first two components for TSO obtained from roasted seeds is presented in Figure 4. The first two principal components account for 73.11% (principal component 1 = 49.74%, principal component 2 = 23.37%) of the total variation. According to our PCA results, TSO obtained after roasting at 130 °C for 20 min showed the most significant alteration. As shown in Table 4, principal component 1 showed positive loadings mainly with the OSI, oil yield, and HMF. The predominantly contributing components of principal component 2 were β-sitosterol, total polyphenols, and phytosterol. Interestingly, the results of PCA analysis showed that the first principal component was closely linked to HMF and that a high concentration of HMF was not an indication of good oil quality. This phenomenon might be due to the accumulation of HMF with the increase in roasting time and temperature. However, in combination with Figure 3, the HMF content contained in TSOs baked at 90, 130, and 170 °C for 10, 20, and 30 min, respectively, showed a generally low level (HMF < 5.0 mg/kg). Therefore, this did not affect the results obtained by PCA.

Our PCA results revealed that the cumulative variance contribution rate of the main components essentially contained most of the information on the quality of TSO, which can be used for comprehensive quality evaluation and classification.

To construct a TSO quality index comprehensive score model, four principal components (*F1*, *F2*, *F3*, *F4*, and *F5*) and their variance contribution rate (*F-total*) were used. The dependent variable *F* is a linear combination of *F1*, *F2*, *F3*, *F4*, and *F5* and is expressed as follows: *F-total* = 49.744% *F1* + 23.369% *F2* + 10.409% *F3* + 5.976% *F4* + 4.811% *F5*.

The score values of TSO prepared with different roasting conditions for each main component are shown in Table 5. The higher score value implies a higher quality of TSO. Our results illustrate that the TSO quality evaluation model scores vary, reflecting a significant difference in the fatty acid components, minor-constituent compositions, and antioxidant parameters of TSO prepared under different conditions. According to the scores and ranking, the comprehensive score of the sample extracted after roasting at 130 °C for 20 min was 0.730, which was higher than the scores of other samples, implying that the sample had the best overall quality.

### 3.13. Hierarchical Cluster Analysis (HCA)

**Figure 5:**
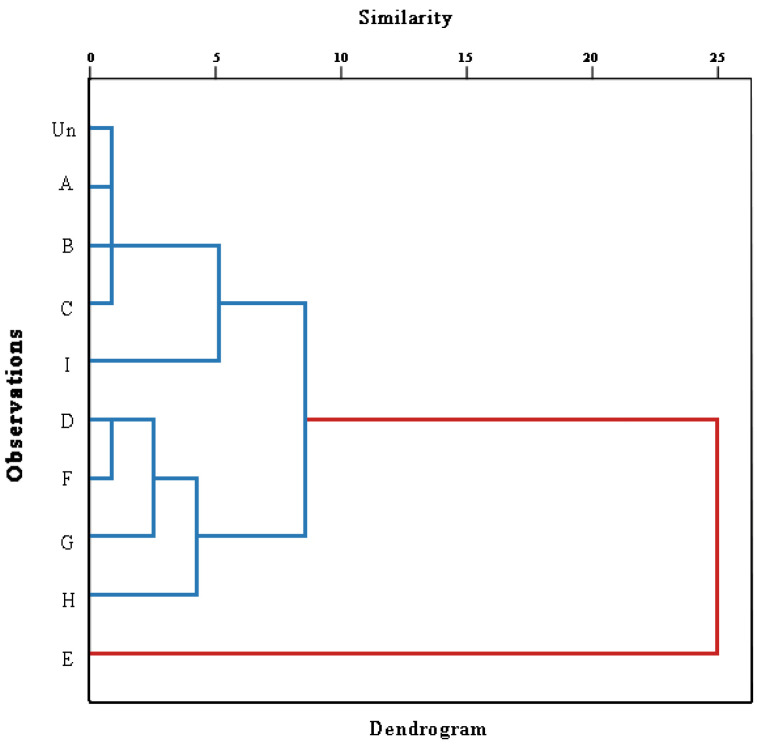
HCA. “Un” represents TSO without roasting; Letters “A–C” represent TSO treated at 90 °C for 10, 20, and 30 min; “D–F” represent TSO treated at 130 °C for 10, 20, and 30 min; and “G–H” represent TSO treated at 170 °C for 10, 20, and 30 min.

Twenty-seven variables with all replicates (*n* = 3) were used for statistical evaluation, including fatty acids, minor component composition, and antioxidant parameters. Based on the analyzed variables, we employed HCA to assess the similarity between the TSO samples. Our results shown in Figure 5 in the form of a tree diagram, illustrate a hierarchy based on the similarity among the samples. The tree structure of the cluster analysis was split into two major sections when a distance threshold of 25 was selected. The TSO of TS roasted at 130 °C for 20 min could be distinctly differentiated from the other samples. According to the results, it was discovered that the roasting process had a considerable effect on the chemical composition and antioxidant capacity of TSO, which indicated that TS roasted at 130 °C for 20 min produced TSO with a better overall quality in terms of its chemical composition, antioxidant activity, and oxidative stability when combined with our PCA results.

## 4. Conclusions

Our study focused on the properties of TSO obtained from TS subjected to roasting at three different temperatures (90, 130, and 170 °C) for 10–30 min. The roasting of TS has a markable effect on the physicochemical properties and fatty acid composition of TSO, which led to a minor decrease in the tocopherol content of the resulting oils. However, it significantly increased the polyphenols, squalene, and phytosterol contents of TSO (*p* < 0.05). Roasting also improved the oxidative stability and antioxidant capacity of TSO, which was presumably due to the enhanced extractability of bioactive compounds and the formation of MRPs. However, some of these biologically active molecules were damaged at temperatures higher than 170 °C. Statistical analysis showed that roasting at 130 °C for 20 min) significantly improved the overall quality of TSO. Future studies will analyze the volatile components of moderately roasted TSOs qualitatively and quantitatively by gas chromatography–mass spectrometry. This will help investigate the effects of temperature and time on the flavor profile of TSOs under moderate roasting, thereby refining the overall research idea. In conclusion, roasting at 130 °C for 20 min is recommended for TS to improve the phenolic contents, quality, properties, and oxidative stability of TSO, which has a wide range of potential applications in the pharmaceutical and food industries.

## Figures and Tables

**Table 1:** Fatty acid profiles of tomato seed oil, unroasted or roasted at 90, 130, and 170 °C.

Compound		Relative Content (%)								
	Unroasted	90 °C			130 °C			170 °C		
		10 min	20 min	30 min	10 min	20 min	30 min	10 min	20 min	30 min
C16:0	13.26 ± 0.13 ^ef^	13.16 ± 0.13 ^f^	13.59 ± 0.16 ^bc^	13.45 ± 0.15 ^cd^	13.32 ± 0.17 ^e^	13.45 ± 0.16 ^cde^	13.53 ± 0.14 ^cd^	13.97 ± 0.11 ^ab^	13.93 ± 0.33 ^a^	13.79 ± 0.12 ^ab^
C18:0	6.11 ± 0.08 ^c^	6.43 ± 0.16 ^bc^	7.08 ± 0.16 ^a^	7.23 ± 0.12 ^a^	6.22 ± 0.16 ^bc^	6.53 ± 0.16 ^b^	7.23 ± 0.12 ^a^	6.61 ± 0.01 ^bc^	7.08 ± 0.28 ^a^	7.17 ± 0.15 ^a^
ΣSFA	19.36 ± 0.20 ^c^	19.59 ± 0.21 ^bc^	20.67 ± 0.23 ^a^	20.67 ± 0.14 ^a^	19.55 ± 0.01 ^bc^	19.97 ± 0.14 ^b^	20.75 ± 0.3 ^a^	20.58 ± 0.1 ^b^	21.02 ± 0.16 ^a^	20.96 ± 0.3 ^a^
C18:1	22.61 ± 0.13 ^b^	22.92 ± 0.12 ^ab^	23.07 ± 0.11 ^a^	23.07 ± 0.01 ^a^	22.70 ± 0.12 ^ab^	22.73 ± 0.14 ^ab^	23.02 ± 0.11 ^a^	22.58 ± 0.16 ^b^	22.80 ± 0.37 ^ab^	23.07 ± 0.11 ^a^
ΣMUFA	22.61 ± 0.13 ^b^	22.92 ± 0.12 ^ab^	23.07 ± 0.11 ^a^	23.07 ± 0.01 ^a^	22.70 ± 0.12 ^ab^	22.73 ± 0.14 ^ab^	23.02 ± 0.11 ^a^	22.58 ± 0.16 ^b^	22.80 ± 0.37 ^ab^	23.07 ± 0.11 ^a^
C18:2	55.59 ± 0.32 ^a^	55.11 ± 0.24 ^bc^	54.04 ± 0.33 ^e^	54.12 ± 0.31 ^e^	55.31 ± 0.23 ^ab^	54.89 ± 0.19 ^cd^	54.00 ± 0.23 ^e^	54.59 ± 0.31 ^d^	54.01 ± 0.29 ^e^	53.80 ± 0.22 ^e^
C18:3	2.44 ± 0.00 ^a^	2.38 ± 0.02 ^ab^	2.22 ± 0.01 ^bc^	2.14 ± 0.01 ^c^	2.44 ± 0.01 ^a^	2.41 ± 0.03 ^ab^	2.23 ± 0.01 ^bc^	2.26 ± 0.03 ^abc^	2.17 ± 0.16 ^c^	2.17 ± 0.02 ^c^
ΣPUFA	58.03 ± 0.36 ^a^	57.49 ± 0.27 ^b^	56.26 ± 0.34 ^d^	56.26 ± 0.33 ^d^	57.75 ± 0.31 ^ab^	57.30 ± 0.22 ^bc^	56.23 ± 0.25 ^d^	56.85 ± 0.3 ^c^	56.18 ± 0.3 ^d^	55.98 ± 0.26 ^d^
MUFA/PUFA	0.39 ± 0.04 ^c^	0.40 ± 0.04 ^b^	0.41 ± 0.07 ^a^	0.41 ± 0.08 ^a^	0.39 ± 0.06 ^c^	0.40 ± 0.07 ^b^	0.41 ± 0.03 ^a^	0.40 ± 0.06 ^b^	0.41 ± 0.03 ^a^	0.41 ± 0.08 ^a^

Notes: Data are presented as the mean ± S.D. Values with unlike letters (a–f) within the same row for a given oil are significantly different (*p* < 0.05). C16:0—palmitic acid; C18:0—stearic acid; C18:1—oleic acid; C18:2—linoleic acid; and C18:3—linolenic acid. ƩMUFA = total monounsaturated fatty acid; ƩPUFA = total polyunsaturated fatty acid; and ƩSFA = total saturated fatty acid.

**Table 2:** Chemical composition of tomato seed oil, unroasted or roasted at 90, 130, and 170 °C.

Compound	Absolute Content (mg/kg)									
	Unroasted	90 °C			130 °C			170 °C		
		10 min	20 min	30 min	10 min	20 min	30 min	10 min	20 min	30 min
α-Tocopherol	7.76 ± 1.28 ^f^	7.89 ± 0.49 ^f^	8.38 ± 0.03 ^f^	11.44 ± 1.43 ^e^	14.53 ± 0.20 ^c^	13.37 ± 0.15 ^d^	12.49 ± 1.30 ^de^	23.42 ± 1.95 ^b^	31.2 ± 1.05 ^a^	31.82 ± 0.43 ^a^
γ-Tocopherol	535.32 ± 1.78 ^a^	526.27 ± 6.98 ^ab^	518.06 ± 7.56 ^b^	498.35 ± 5.27 ^cd^	513.15 ± 4.29 ^bcd^	512.59 ± 4.86 ^bcd^	498.73 ± 2.76 ^de^	485.12 ± 3.12 ^e^	453.66 ± 5.08 ^f^	446.89 ± 0.28 ^f^
δ-Tocopherol	213.52 ± 0.16 ^a^	210.74 ± 2.71 ^ab^	209.33 ± 4.61 ^abc^	196.01 ± 0.91 ^e^	205.68 ± 1.39 ^bcd^	201.81 ± 0.63 ^cd^	190.16 ± 2.41 ^fg^	194.35 ± 1.49 ^ef^	185.69 ± 0.74 ^g^	163.71 ± 0.82 ^h^
Total-Tocopherol	758.53 ± 0.89 ^a^	748.16 ± 8.41 ^ab^	738.06 ± 5.72 ^ab^	707.54 ± 4.61 ^d^	734.59 ± 0.26 ^bc^	729.50 ± 0.91 b^c^	702.45 ± 3.24 ^d^	704.93 ± 1.68 ^d^	672.11 ± 4.22 ^e^	643.72 ± 1.39 ^f^
β-Sitosterol	1600.66 ± 23.75 ^de^	1610.56 ± 27.19 ^de^	1626.30 ± 33.64 ^d^	1584.94 ± 14.26 ^e^	1687.46 ± 12.58 ^bc^	1788.64 ± 4.90 ^a^	1708.11 ± 7.56 ^b^	1679.44 ± 13.81 ^c^	1586.95 ± 22.63 ^e^	1590.79 ± 4.20 ^e^
Stigmasterol	369.21 ± 1.87 ^d^	390.48 ± 9.61 ^c^	411.12 ± 2.77 ^a^	365.13 ± 0.51 ^e^	359.04 ± 7.94 ^e^	408.52 ± 1.08 ^ab^	404.52 ± 1.08 ^b^	361.95 ± 7.55 ^e^	343.38 ± 5.61 ^fg^	345.96 ± 3.98 ^f^
Δ5-Avenasterol	216.00 ± 3.25 ^f^	216.84 ± 1.56 ^f^	218.03 ± 0.89 ^ef^	221.12 ± 1.39 ^de^	230.88 ± 0.82 ^b^	244.42 ± 0.48 ^a^	223.63 ± 2.93 ^d^	226.82 ± 1.26 ^c^	234.02 ± 4.10 ^b^	221.83 ± 0.66 ^d^
Cholesterol	451.11 ± 2.15 ^b^	439.97 ± 0.89 ^c^	421.43 ± 3.19 ^d^	413.26 ± 0.80 ^e^	389.52 ± 4.34 ^f^	450.01 ± 4.68 ^b^	456.93 ± 2.20 ^a^	413.09 ± 1.43 ^e^	394.90 ± 2.28 ^f^	390.74 ± 2.96 ^f^
Campesterol	93.25 ± 1.36 ^a^	91.76 ± 3.16 ^ab^	84.95 ± 3.54 ^c^	81.21 ± 1.81 ^d^	81.21 ± 1.81 ^d^	88.57 ± 6.02 ^c^	89.37 ± 4.29 ^bc^	81.31 ± 6.61 ^d^	80.04 ± 0.84 ^de^	81.08 ± 2.11 ^d^
Cycloartenol	59.33 ± 6.52 ^b^	56.21 ± 0.79 ^de^	56.84 ± 0.48 ^cd^	55.42 ± 2.03 ^de^	58.73 ± 0.62 ^bc^	57.15 ± 2.29 ^cd^	61.46 ± 0.76 ^a^	59.15 ± 2.24 ^b^	54.32 ± 1.22 ^e^	58.41 ± 4.80 ^bc^
Phytosterol	2789.56 ± 19.34 ^b^	2805.82 ± 23.69 ^b^	2818.67 ± 21.15 ^b^	2721.08 ± 17.58 ^c^	2806.84 ± 20.73 ^b^	3037.31 ± 28.57 ^a^	2487.09 ± 17.65 ^e^	2821.76 ± 19.41 ^b^	2693.61 ± 30.94 ^d^	2216.99 ± 8.27 ^f^
Squalene	5.06 ± 0.51 ^g^	5.94 ± 0.22 ^g^	7.36 ± 0.08 ^ef^	10.11 ± 0.34 ^c^	8.12 ± 0.04 ^de^	13.10 ± 0.72 ^a^	11.48 ± 1.11 ^b^	6.82 ± 0.35 ^f^	8.32 ± 0.37 ^d^	10.62 ± 0.24 ^bc^
Total polyphenols ^A^	22.95 ± 1.13 ^d^	25.43 ± 3.22 ^bc^	25.14 ± 0.19 ^c^	23.71 ± 2.48 ^d^	24.66 ± 1.36 ^c^	26.67 ± 0.41 ^a^	26.52 ± 1.53 ^ab^	22.90 ± 1.25 ^d^	23.61 ± 2.09 ^d^	22.37 ± 3.10 ^d^

Notes: Values with unlike letters (a–g) within the same row for a given oil are significantly different (*p* < 0.05). “A”: unit is mg GAE/100 g.

**Table 3:** Oxidative stability index (h) and antioxidant capacity (μmol TE/100 g) of tomato seed oils.

Roasting Temperature	Roasting Time	OSI (h)	DPPH (μmol TE/100 g)	ABTS (μmol TE/100 g)	FRAP (μmol TE/100 g)
Unroasted	—	5.35 ± 0.03 de	43.17 ± 1.96 f	51.261 ± 0.19 de	36.39 ± 0.33 e
90 °C	10 min	4.82 ± 0.04 e	56.96 ± 2.43 c	49.791 ± 0.21 e	36.90 ± 0.83 e
	20 min	5.71 ± 0.19 d	59.87 ± 1.61 a	52.60 ± 3.84 d	44.78 ± 1.97 a
	30 min	6.66 ± 0.11 c	57.99 ± 3.11 b	53.48 ± 0.09 cd	43.17 ± 0.95 b
130 °C	10 min	4.83 ± 0.06 e	46.79 ± 0.57 e	54.87 ± 3.11 c	43.17 ± 2.16 b
	20 min	6.73 ± 0.20 c	59.78 ± 2.68 a	59.95 ± 1.85 a	43.25 ± 0.42 b
	30 min	7.12 ± 0.09 b	55.40 ± 1.12 cd	55.03 ± 0.07 bc	44.65 ± 2.25 a
170 °C	10 min	4.67 ± 0.16 e	48.17 ± 2.96 d	48.23 ± 0.14 f	37.36 ± 1.44 d
	20 min	7.36 ± 0.28 a	59.38 ± 1.50 a	52.92 ± 2.70 d	42.41 ± 2.68 c
	30 min	7.07 ± 0.27 b	59.69 ± 3.75 a	56.16 ± 2.72 b	44.06 ± 0.93 ab

Notes: Values with unlike letters (a–f) within the same column for a given oil are significantly different (*p* < 0.05). “—”: treated without roasting.

**Table 4:** Eigenvector, eigenvalues, variance contribution rates, and cumulative contribution rates of principal component analysis.

Variable	Components	F1	F2	F3	F4	F5
X_1_	Total polyphenols	0.085	0.868	−0.235	0.243	−0.071
X_2_	Oil yield	0.947	−0.019	−0.068	0.013	−0.02
X_3_	POV	0.752	−0.573	0.175	0.266	0.031
X_4_	AV	−0.925	−0.153	−0.128	−0.133	0.164
X_5_	ABTS	0.007	0.641	−0.075	0.647	0.276
X_6_	FRAP	−0.511	0.402	−0.382	0.396	0.296
X_7_	DPPH	0.251	−0.392	−0.488	0.634	−0.307
X_8_	BI	0.663	−0.589	0.296	0.105	0.149
X_9_	HMF	0.913	−0.012	0.029	0.012	−0.109
X_10_	C16:0	−0.273	−0.242	0.792	0.001	0.163
X_11_	C18:0	−0.835	−0.047	0.477	0.155	−0.11
X_12_	C18:1	−0.788	0.03	−0.004	0.3	−0.384
X_13_	C18:2	0.785	0.07	−0.574	−0.165	0.117
X_14_	C18:3	0.779	0.307	−0.49	−0.179	0.033
X_15_	Stigmasterol	0.636	0.602	0.382	0.061	0.233
X_16_	β- sitosterol	−0.087	0.879	0.331	−0.049	−0.319
X_17_	Δ5-Avenasterol	0.709	0.494	0.017	−0.28	−0.376
X_18_	Cholesterol	0.681	0.577	0.362	0.063	0.184
X_19_	Campesterol	0.475	0.725	0.14	0.101	0.446
X_20_	Cycloartenol	0.721	0.385	0.24	0.077	−0.455
X_21_	Squalene	0.741	0.559	−0.318	−0.088	−0.073
X_22_	Phytosterol	0.308	0.862	0.372	−0.018	−0.122
X_23_	OSI	0.96	−0.059	0.071	0.152	0.011
X_24_	Total tocopherols	−0.884	0.426	−0.001	−0.181	0.029
X_25_	α-Tocopherol	0.789	−0.441	0.39	0.126	−0.022
X_26_	γ-Tocopherol	−0.847	0.444	−0.159	−0.232	−0.003
X_27_	δ-Tocopherol	−0.899	0.4	0.071	−0.046	0.09
Characteristic value		13.928	6.5434	2.915	1.673	1.347
Variance contribution rate (%)		49.744	23.369	10.409	5.976	4.811
Cumulative variance contribution rate (%)		49.744	73.113	83.522	89.499	94.31

Notes: C16:0—palmitic acid; C18:0—stearic acid; C18:1—oleic acid; C18:2—linoleic acid; and C18:3—linolenic acid.

**Table 5:** Quality evaluation of tomato seed oil from different roasting conditions.

Samples	*F1*	*F2*	*F3*	*F4*	*F5*	*F-Total*	Sequence
Unroasted	−1.25208	−0.71214	0.37288	−1.99261	0.80049	−0.831	10
90 °C, 10 min	−1.18462	−0.27328	−0.49983	0.60111	−0.25088	−0.681	9
90 °C, 20 min	−0.96374	−0.00551	−0.68179	1.38102	−0.17007	−0.477	8
90 °C, 30 min	−0.14641	−0.0935	−1.43871	−0.14616	0.61412	−0.224	7
130 °C, 10 min	−0.3834	0.83876	1.00741	0.21699	0.70703	0.157	5
130 °C, 20 min	0.62083	2.021	0.30154	−0.97784	−0.50309	0.730	1
130 °C, 30 min	0.73695	0.88904	−0.97687	0.30729	−0.30051	0.477	3
170 °C, 10 min	−0.16318	−0.74519	1.29621	0.16185	−2.14675	−0.214	6
170 °C, 20 min	1.02529	−0.49369	1.34507	1.09458	1.5352	0.674	2
170 °C, 30 min	1.71037	−1.42549	−0.7259	−0.64624	−0.28554	0.390	4

## Data Availability

The original contributions presented in the study are included in the article, further inquiries can be directed to the corresponding author.
